# University of Pennsylvania

**Published:** 1897-07

**Authors:** 


LEONARD PEARSON.

---

### COMMENCEMENT EXERCISES.

### VETERINARY DEPARTMENT OF THE UNIVERSITY OF PENNSYLVANIA.

THE closing exercises of this department were held in conjunction with the several other departments on Wednesday, June 9th, at 11 A.M. The members of the graduating class marched with the other graduates from the college to the Academy of Music. The procession, headed by the City Troop and followed by the municipal band, led off with the Governor of the State, Mayor of the city, faculties of the various departments, and graduates. Of the class of nineteen, some seventeen attained the necessary average to win their diplomas. The degree of V.M.D. was conferred upon them by Provost Harrison, after an appropriate prayer by the Most Reverend Archbishop Ryan. A very excellent and timely address on the duties of citizens was delivered by Hon. Wayne MacVeagh, ex-Ambassador to Italy, to those who received degrees from this honored institution. The exercises were interspersed with excellent music.

List of graduates: Raymond Barber, James Beatty, Henry Bower, Jose Traugot Hernsheim, Louis Amos Klein, Abraham Lincoln Lehman, Augustus Nathaniel Lushington, Henry Marshall, John Wood Montague, Edward William O'Connor, Edward Martin Ranck, William George Shaw, William Henry Smith, Jr., William J. Storm, Archibald Hay Wallace, Ernest Aarons White, Lloyd Zaner.

The first prize of one hundred dollars, known as the J. B. Lippincott prize, was awarded Dr. Louis A. Klein, of the graduating class. A prize of an écraseur, given to the member of the junior class passing the best examination, was won by Mr. Guy E. Chesley. A group-picture of the graduating class was prepared for the college and the several members of the class.

On the evening of June 8th the Alumni Association held their annual reunion at the Aldine Hotel, where, grouped around a number of small tables, were to be noted, among others, Professors Adams and Harger, Drs. H. P. Turner, Schrieber, C. J. Marshall, Christman, Houldsworth, McCray, Senseman, John Pearson, and of the '97 graduates Messrs. Barber, Beatty, Hernsheim, Lushington, Henry Marshall, Shaw, Montague, Wallace, and Ranck. President Adams, in an informal manner, called the meeting to order, and Dr. Clarence J. Marshall reported for the Committee of Arrangements. The choice of officers for the ensuing year resulted in the selection of Dr. A. F. Schrieber, President, who enlivened the occasion with several songs and stories; Vice-President, Dr. C. J. Marshall; and Secretary-Treasurer, Dr. Wm. G. Shaw. After thanking the officers and instructors, the gathering dispersed at midnight.

The veterinary medical association of the students met at 4 P.M. on Wednesday, June 9th, in the college hall; about fifteen members were present. The librarian announced the receipt of additional books. A sum of money was appropriated to complete the certificates of membership and their delivery to the fourteen members who had complied with the requirements, and to whom they were awarded. The officers for the year were: Honorary President, Rush Shippen Huidekoper, New York; Honorary Secretary, Leonard Pearson, Philadelphia; President, L. A. Klein; Vice-President, E. M. Ranck; Secretary, John E. Spindler, Class '96; Treasurer, James Beatty; Executive Committee, E. M. Ranck and Wm. G. Shaw, Class '97; Ernest Spaeth and B. Kirby, Class of '98; C. F. Keiter, Class of '99; Librarian, Stephen Blunt, Class '98.

A class-meeting of the graduates of 1897 was held on commencement day, where, after receiving reports from the several class committees, the officers, consisting of President, Wm. G. Shaw; Vice-President, Lloyd Zaner; Secretary, Wm. H. Smith, Jr.; Treasurer, Edward O'Connor, with the other members of the class, chose as permanent Secretary, Dr. Henry Marshall, of Georgetown, Delaware, to whom all matters concerning the individual members of the class should be sent, and from whom information could be obtained pertaining to the Class of '97.

